# Prerequisites of Preparedness against Earthquake in Hospital System: A Survey from Iran

**DOI:** 10.5539/gjhs.v6n2p237

**Published:** 2014-02-21

**Authors:** Sayyed Morteza Hosseini Shokouh, Mina Anjomshoa, Seyyed Meysam Mousavi, Jamil Sadeghifar, Bahram Armoun, Aziz Rezapour, Mohammad Arab

**Affiliations:** 1 Health Management Research Center & School of Public Health, Baqiyatallah University of Medical Sciences and School of Public Health, Tehran University of Medical Sciences, Tehran, Iran; 2 Research Center for Health Services Management, Institute for Futures Studies in Health, Kerman University of Medical Sciences, Kerman, Iran; 3 Research Center for Modeling in Health, Institute for Futures Studies in Health, Kerman University of Medical Sciences, Kerman, Iran; 4 Students’ Scientific Research Center, School of Public Health, Tehran University of Medical Sciences, Tehran, Iran; 5 Health Management and Economics Research Center, Iran University of Medical Sciences, Tehran, Iran; 6 Hospital Management Research Center, Iran University of Medical Sciences, Tehran, Iran; 7 School of Public Health & Health Management Research Center, Baqiyatallah University of Medical Sciences, Tehran, Iran; 8 School of Public Health, Tehran University of Medical Sciences, Tehran, Iran

**Keywords:** preparedness, earthquake, disaster, hospital, Iran

## Abstract

**Background and Objective:**

Considering the history of frequent, and severe, earthquakes in Iran and the importance of health care service delivery by hospitals in these cases, having a plan to deal with disasters should be considered a priority. The aim of this study was the observance of preparedness prerequisites against earthquake in hospitals affiliated with Shahid Beheshti University of Medical Sciences (SBUMS) and its relationship with demographic and organizational characteristics.

**Methods:**

This was a cross- sectional study that was conducted in 15 hospitals affiliated with SBUMS, Iran in 2012. Data were collected using observation of documents and questionnaire consists of 138 questions in 8 dimensions. The content validity and reliability were confirmed. Data analysis was performed with descriptive statistic, t-test and ANOVA.

**Results:**

Results showed that 86.7% of hospitals were in good preparedness level, with the average 85.9 ± 15.5. The maximum and minimum level of preparedness was related to mitigation of construction hazards (56.6 ± 35.6) and support of vital services (97.2 ± 6.0) dimensions, respectively. According to the results, there was a significant statistical difference between mean preparedness and safety of equipment and hazardous materials, hospital evacuation and field treatment, hospital environmental health proceedings, hospital curriculum programs and support of services dimensions with management experience (P<0.05).

**Conclusion:**

Although results corroborate that preparedness prerequisites against earthquake are in good level but attention to the weaknesses mitigation of construction hazards dimension and strengthening these prerequisites, which have obvious impacts on the structural vulnerability of hospitals and adjacent buildings in earthquakes, have been proposed.

## 1. Introduction

Natural disasters such as earthquakes, floods, volcanic flows, tsunamis, storms and lightning and also man-made disasters such as terrorist attacks, chemical explosions, industrial accidents, collapsing buildings, transportation accidents and wars, potentially, have great life and financial risks (Pengfei, Santhosh, Jomon, & Li, 2010). Earthquake as the most destructive natural disaster usually occurs in a completely random form without any previous warning and forecasting and can happen at any time, day or night, and any year (Mehta, 2006). On average, 70 to 75 damaging earthquakes occur throughout the world, each year (American Medical Association, 2005).

Iran ranks tenth in the disaster prone countries in the world and fourth in Asia; so out of 40 types of known disasters in the world, occurrence of 31 of them has been reported in Iran, which dedicates a total of 6 percent of all accidents and adverse natural disasters in the world. Iran as a result of standing on the seismic belt of the Alp-Himalaya is known as one of the permanent victims of earthquake, which is the most common cause of death, related to natural disasters (Pourmalek, Ardalan, Russel, & Mohammadi, 2006). According to the statistics, out of world’s 5great earthquakes, occurred from 1990 to 2005, two were related to Iran, and most of the casualties have been related to these two cases (Sztajnkrycer, 2005). Approximately, every decade large earthquakes occur in different parts of the country and cause a lot of distraught and mourning, but the note in this case is the location of Tehran as the capital of Iran, with a population of more than 10million people, in an area with high possible risk of earthquake (Pourmalek et al., 2006; Nateghi, 2000). The metropolis of Tehran is not only safe from earthquakes, but for years has been waiting for a devastating earthquake with a power above seven on the Richter scale. The main reason for earthquake in Tehran is 15 faults in this zone, which only three of them alone have the potential to create an earthquake with power more than seven on the Richter scale (Nateghi, 2000).

Despite the fact that natural disasters can^’^t be predicted with any degree of certainty, it is essential for organizations that are burdened with the task of health service delivery, to design the urgent preparedness programs in order to reduce the losses as much as possible (Kaji, Koenig, & Lewis, 2007). Preparation of the health sector as the institution providing health service is necessary and vital in reducing mortality and physical injuries resulting from accidents, crisis and emergency situation, significantly (Gupta & Kant, 2004) and efficient organizing and management of the health care centers in the time of crisis has great influence on optimal and efficient functionality of these institutions (Bulut et al., 2005).

Health care centers, as the main organizations involved in the event of disasters, especially earthquakes, require a codified disaster preparedness program (Halpern & Chaffee, 2005; Rubin, 2006). For health centers, specifically hospitals, having such a program are crucial, particularly considering its function and the first place of accident victim’s referrals (Krajewski, Sztajnkrycer, & Baez, 2005). Regardless of type and capacity, hospitals should have a disaster preparedness program (Hersche & Wenker, 2000).

The analysis of the effects of natural disasters has shown that most hospitals are not prepared to deal with natural disasters’ problems. For example, results of a study on “the simultaneous victim’s admission capacity in a normal situation” in Tehran hospitals showed that the mean admission number was 8 and the maximum was 60 people. In other words, with about 100 hospitals in Tehran, admission of injured people in all hospitals was estimated to be 800 people, while it is estimated that stroke of a severe earthquake in Tehran will leave hundreds of thousands of wounded (Hosseini Shokouh, Arab, Rahimi, Rashidian, & Sadr Momtaz, 2008).

Preparedness of hospitals against disasters originates from multiple and complex factors, which formulate hospital disaster plan is one of the most important factors. Formulating such program is an important priority of any hospital board of directors and managers, which is the first step for creating hospital preparation in disasters (Mehta, 2006; Peltz et al., 2006; McGlown, 2004). Experience of other countries has shown that the hospitals that had a plan and practice it regularly, have suffered less damage during the disasters (Katrina, 2003; Vinson, 2007). Of course, the existence of a program is not sufficient alone, but also readiness of hospital for program implementation is definitive, and this program testable in practice and with periodic exercises and this should be noted that the preparedness is not cross sectional and not subject to a particular time and place and requires permanent practices and attitudes, and must be achieved before disasters, cause the time of disaster is certainly not the time to learn by trial and error (Emami et al., 2005). In addition, the program must be dynamic and continually gets tested, revised and updated (Remmen, 2005). It should be noted that the success of the proposed plan for disaster preparedness and coping with unexpected events such as hospital emergency incident command system (HEICS or HICS) in the developed countries is such that nowadays hospitals in these countries could not be evaluated without such plan.

Now, more than a decade has passed since the executive earthquake instructions were issued to hospitals as “Planning Guide for Hospitals to deal with quake”. This guide provides a planning process for managers to help them organize their activities and formulate hospital’s specific programs to deal with earthquakes. In this regard, the aim of this study was the observance of earthquake preparedness prerequisites in hospitals affiliated with Shahid Beheshti University of Medical Sciences (SBUMS) and its relationship with demographic and organizational characteristics.

## 2. Methods

This was a cross-sectional study that was conducted in 2012 (April to September). Research environment includes 17 hospitals affiliated with SBUMS (one of the three major universities in the field of medical sciences in Tehran, the capital of Iran). The data were obtained from information resources, including hospitals’ chairman, manager, head of nursing, training liable, the facility liable, medical records liable, hygiene liable and the manager of hospital disaster committee in all of the hospitals.

Data about structural and non-structural preparedness against earthquake in hospitals were collected via the researcher made questionnaire in 8 planning dimensions including: 1) safety of equipment and hazardous materials (34 questions); 2) mitigation of construction (8 questions); 3) hospital evacuation and field treatment (19 questions); 4) Necessary medical and nonmedical equipment and consumable goods (15 questions); 5) hospital environmental health proceedings (16 questions); 6) hospital curriculum (18 questions); 7) disaster plan management (12 questions) and 8) planning of support of critical services (17 questions), through interview and observation of correspondences and documentations. Another questionnaire was used for gathering of hospital manager’s demographic profiles and organizational characteristics of hospitals.

Scale of questions on the questionnaire was Yes or No. This questionnaire consists of 138 questions, which overall score would be 138 if the answers to all the questions are “Yes”. Finally, according to the earned scores, situation of earthquake preparedness in the each planning dimensions was categorized into three groups: A) weak (zero to less than 50%); B) moderate (50% to less than 75%) and C) good (75% to 100%). The validity of the questionnaire had been confirmed in similar studies (Hosseini Shokouh et al., 2008; Mohabati, 2005). The Cronbach’s alpha coefficient was used to determine the reliability (α= 82.8%), that this coefficient demonstrated the internal consistency and homogeneity of the questionnaire.

According to the type of study, do not gain any formal ethical approval from the studied University’s Research Ethics Committee. Normally, questionnaires need not a written consent form. So, the participants consented verbally. The return of the completed questionnaire demonstrates their consent to participate in the study. Participants volunteered for the study and visited by the researchers at their work place. Researchers were committed to the ethical principles regarding the publication of the results, so the results will be presented in general form and not separately for each hospital.

Data were entered and processed using the Statistical Package for the Social Sciences (SPSS) software, the version 18, ANOVA and T-test.

## 3. Findings

Out of 17 hospitals affiliated with SBUMS, data were collected from 15 hospitals (RR=88.2%). Results show that 60% of the hospitals under the survey were general, 66.7% had teaching activities, 46.7% had more than 10 active departments and 73.3% of the hospitals were identified as a first degree in the last evaluation. Also, the results showed that all managers of the hospitals were married and have been hired officially, 60% of them have a Master’s degree and higher and 86.7% of them were educated in the fields of non-managerial, although all of them have been trained in hospital management and disaster management’s short training program (Table 1).

**Table 1:** Frequency distributions of hospital organizational characteristics and manager’s demographic profiles

Hospitals characteristics				Hospitals Managers characteristics			
**Variables**		**n**	**f**	**Variables**		**N**	**f**
**Type of hospital**	General	9	60	**Mariette statues**	Married	15	100
	Specialize	6	40		Single	0	0
**Hospital activity**	Teaching	10	66.7	**Education level**	MSc and higher	9	60
	Non-teaching	5	33.3		BSc and lower	6	40
**Number of active departments**	10 and less than 10	7	46.7	**Age**	45 and lower	9	60
	More than 10	8	53.3		Higher than 45	6	40
**Evaluation degree**	One	11	73.3	**Educational field**	Management	1	6.7
	Two	3	20		Others	13	86.7
**Number of active beds**	200 and less than 200	9	60	**Short training program**	Yes	15	100
	More than 200	6	40		No	0	0
**Bed Occupancy Rate (BOR)**	80 and less than 80	13	86.7	**Work experience**	10 and less than 10	9	60
	More than 80	2	13.3		More than 10	6	40
**Buildings’ age**	30 and less than 30	5	33.3	**Management experience**	5 and less than 5	12	80
	More than 30	10	66.7		More than 5	3	20
**Number of staff**	300 and less than 300	4	26.7	**Type of hire**	Official	15	100
	300 to 600	6	40		Others	0	0
	More than 600	5	33.3	

Results showed that 86.7% of hospitals were in good levels of preparedness with an average of 50.1 ± 94.8, and also, maximum and minimum levels of preparedness in 8 investigated preparedness dimensions were in mitigation of construction hazards dimension with an average of 56.6 ± 35.6 and support for critical services dimension with 97.2 ± 6.0, respectively (Table 2).

**Table 2:** Frequency distribution, mean, standard deviation and the level of hospitals preparedness, according to the preparedness dimensions against earthquake

Preparedness dimensions against Earthquake	Level of preparedness frequency distribution						Mean	SD	Level of preparedness
	Weak		Moderate		Good				
	n	f	n	f	n	f			
Safety of equipment and hazardous materials	1	6.7	4	26.7	10	66.7	81.2	20.7	Good
Construction mitigation	7	46.7	2	13.3	6	40	56.6	35.6	Moderate
Hospital evacuation and field treatment	1	6.7	0	0	14	93.3	91.2	22.9	Good
Necessary medical and nonmedical equipment and consumable goods	4	26.7	0	0	11	73.3	79.1	35.0	Good
Hospital environmental health proceedings	1	6.7	4	26.7	10	66.7	83.3	19.5	Moderate
Hospital curriculum program	1	6.7	0	0	14	93.3	93.3	19.0	Good
Disaster plan management	2	13.3	0	0	13	86.7	95.6	11.4	Good
Support for critical services	0	0	0	0	15	100	97.2	6.0	Good
Overall	1	6.7	1	6.7	13	86.7	85.9	15.5	Good

The results indicate that depending on the type of hospitals (general and Specialized) there were differences between the average of hospital preparedness planning dimensions, in hospital curriculum, support of critical services and disaster plan management, the t-test also reveals this is as statistically significant (p<0.05). Furthermore, there was a statistically significant difference between the average of hospital’s preparedness in necessary medical and nonmedical equipment and consumable goods dimension with manager educational field (p<0.05) (Table 3).

**Table 3:** Mean of preparedness dimensions against earthquake in significant variables according to t-test p-value

Preparedness dimensions against Earthquake	Variables		n	Mean	P- value
Safety of equipment and hazardous materials	BOR	80 and less than 80	13	84.1	0.0001
		More than 80	2	62.1	
	Management experience	5 and less than 5	12	82.8	0.001
		More than 5	3	74.7	
Hospital evacuation and field treatment	BOR	80 and less than 80	13	96.7	0.0001
		More than 80	2	55.2	
	Management experience	5 and less than 5	12	96.4	0.0001
		More than 5	3	70.1	
Necessary medical and nonmedical equipment and consumable goods	Educational field	Management	2	96.6	0.018
		Others	13	76.4	
Hospital environmental health proceedings	Management experience	5 and less than 5	12	80.2	0.044
		More than 5	3	95.8	
Hospital curriculum program	Type of hospital	General	9	88.8	0.04
		Specialized	6	100	
	BOR	80 and less than 80	13	97.8	0.0001
		More than 80	2	63.8	
	Management experience	5 and less than 5	12	97.6	0.0001
		More than 5	3	75.9	
Support for critical services	Type of hospital	General	9	92.8	0.005
		Specialized	6	100	
	BOR	80 and less than 80	13	97.7	0.019
		More than 80	2	82.3	
	Management experience	5 and less than 5	12	97.5	0.028
		More than 5	3	88.2	
Disaster plan management	Type of hospital	General	9	95.3	0.001
		Specialized	6	100	
Overall	BOR	80 and less than 80	13	88.4	0.0001
		More than 80	2	69.9	
	Management experience	5 and less than 5	12	87.4	0.0001
		More than 5	3	79.9	

According to the results, there were differences between the average of hospital’s preparedness in mitigation of construction hazards dimension with hospital buildings’ age, that the ANOVA showed a statistically significant (p<0.05). The Tukey complementary test suggests that, this difference in both cases relates to the hospital buildings’ age over than 30 years, that is, hospitals with higher building age have higher preparedness. Also, the results showed that there were no differences between the average of hospital’s preparedness with the number of fixes and active beds, number of active departments, evaluation degree, number of staff and work experience, which the ANOVA revealed was not statistically significant(p>0.05). Furthermore, there were no differences between the average of hospital’s preparedness with hospital’s type, activity, age, educational level and field of managers, as the t-test showed that this difference is not statistically significant (Table 4).

**Table 4:** Mean value of preparedness dimensions against earthquake in significant variables according to ANOVA p-value

Dimensions against Earthquake	Variables		n	Mean	P- value
Hospital evacuation and field treatment	Number of active beds	200 and less than its	9	95.3	0.04
		Between 200 and less than 400	4	100
		More than 400	2	55.2
Construction mitigation	Buildings’ age	30 and less than its	5	20	0.007
		Between 30 and less than 60	7	75
		More than 60	3	75
Hospital environmental health proceedings	Number of active Department	5 and less than its	4	78.1	0.02
		Between 5 and less than 10	4	65.6
		More than 10	7	96.4
Hospital curriculum program	Number of active beds	200 and less than its	9	96.9	0.047
		Between 200 and less than 400	4	100
		More than 400	2	63.8

## 4. Discussion

In our study, the status of preparedness planning and management against earthquakes were examined at hospitals affiliated with SBUMS, in eight dimensions, including: safety of equipment and hazardous materials, mitigation of construction hazards, hospital evacuation and field treatment, necessary medical and nonmedical equipment and consumable goods, hospital environmental health proceedings, hospital curriculum, disaster plan management and support of critical services. According to the results, 86.7 percent of the hospitals were in good level with a mean value equal to 85.9, such that the lowest level of preparedness related to mitigation of construction hazards dimension, in addition the highest level related to disaster plan management dimension.

In the case of safety of equipment and hazardous materials dimension, about half of the hospitals had no action plan about strengthen the glasses by glue and glass opaque, preventing scattering of the glass in the case of disaster occurrence, formulate a particular treatment program in the case exposure of personnel to radioactive materials and chemical pollutants after a disaster, continuous control of fires alarm systems and the firefighting systems. About one-third of the hospitals had not carried out any actions such as holding a contract with a construction engineering company for evaluation of hospital buildings after a quake, measurements to determine hospital building’s vulnerability, actions to determine the risk of hospital’s structure for public security, to determine the possibility of strengthening the buildings and its costs and preparation of the list of high risk structures to use in evacuation program and vulnerability assessment. In hospital evacuation and field treatment dimension, all investigated hospitals were in good conditions, only about one-third of them had not done anything about establishment of mobile elevators - power operated or manual- in hospital’s courtyard, for patients who are not able to move. About one-third of hospitals had not taken any measurements about sanitation of sewer collection after earthquake, formulating a healthy transfer of the collected sewage in hospital, from production to treatment places and developing a program to evaluate the biological quality (total coli forms and E. coli) of hospital’s water after an earthquake. All of these actions relate to hospital’s environmental health proceedings dimension.

In a research about disaster preparedness of hospitals in Turkey, 92.8% of 233 studied hospitals had plan to deal with the crisis, as well as 58.4% of hospitals at least once a year organizes an annual maneuver (Bulut et al., 2005). Afkar et al. (2013) showed that hospitals of Guilan province in Iran were prepared against earthquake, moderately. In the investigation conducted by Shojaei et al. (2009), which was done in selected hospitals affiliated with Iran University of Medical Sciences, highest and lowest scores for hospital’s readiness were equal to 65.6 and 54.3, respectively. Arab (2007) determined the score of hospitals preparedness affiliated with Tehran University of Medical Sciences to be 49.5%, which was in low level. Preparedness level of the Iran University of Medical Sciences’ hospitals against earthquake in Hosseini Shokouh (2008) study showed that, on the whole, 28.6% of the hospitals were at a weak level of preparedness, 61.9% were at a moderate level and 9.5% were on a good preparedness level. In a study of U.S.A. hospitals in 2003, only 22% of hospitals were prepared to deal with the crisis (Murphy, 2004). Richard and Catharine (2005) have stated that 97.3% of hospitals had plans to cope with natural disasters such as earthquakes, floods and etc. According to a study that was conducted in the Netherlands in 2002, 74% of general hospitals were not completely prepared to face disasters (Remmen, 2006). A study in Los Angeles showed 64% of hospitals had plans for dealing with a Mass Casualty Incident (Kaji et al., 2007).

As the results of our study and its comparison with the results of national and international studies have shown, two reasons can be found to justify the differences in hospital preparedness: first, in some studies, hospitals investigated were in a good situation, because they had preparation programs to deal with the crisis, whereas this is different from practical aspects of preparedness. Second, tools for assessment of preparedness level in hospitals, with different types of crises, were different and therefore it yields conflicting results.

Perhaps one of the reasons for the high level of preparedness in investigating hospitals, in comparison with other studies, was the short term training programs in hospital management and disaster management taken by all hospital managers. Therefore, it seems desirable to develop a systematic and consistent implementation of such short-term training programs in the field of crisis management. Also, beside hospital administrators, it is better for other officials and experts to participate in the training programs, to expand the effective scope of educational programs and reach a balance and increase in the hospital preparedness to a much greater extent (Nelson et al., 2005).

According to the results, there was a statistically significant difference between the mean value of hospital’s preparedness in safety of equipment and hazardous materials, evacuation and field treatment, environmental health proceedings, curriculum and support of critical services with management experience. Therefore, the total mean score of preparedness and also hospital’s preparedness in the safety of equipment and hazardous materials, evacuation and field treatment, curriculum and support of critical services dimensions were higher in studying hospitals, that the managers had 5 and less than 5 years management experience, while the level of preparedness in hospital environmental health proceedings was higher in hospitals, which the managers had more than 5 years management experience. Also, there were differences between the mean value of preparedness in hospital evacuation and field treatment and hospital curriculum program with hospital bed numbers. Hence, preparedness in hospitals, which has a bed number between 200 and 400, was higher than others, in each of the two components.

There was a statistically significant difference between the average values of hospital preparedness planning components in mitigation of construction hazards with hospitals’ building age; that is, preparedness was higher in hospitals which the buildings were more than 30 years old. Also, there was a statistically significant difference between mean values of preparedness in hospital’s environmental health proceedings planning component with the number of active departments, which was higher in hospitals that has been more than 10 active departments.

## 5. Limitation

This study had some potential limitations that may affect the results. The study is limited to hospitals of a single university. The results of the present study were solely the result of a research and possibly more specialized investigation of these hospital^’^s preparedness against earthquake will show the most precise results. This study has been designed based on the instructions of Iran’s Ministry of Health for the preparedness of hospitals against earthquake. Therefore, generalizability of results can be considered as a limitation of the present study. An insufficient literature, particularly in international level for comparison with current findings with them is another limitation in this study. The best important strengths in this study are related to interesting topics and data gathering methods. The used questionnaire in this study for assessing preparedness of hospitals against earthquakes is a good tool, so that many of the key point of this tool, can be found in Hospital Emergency Incident Command System (HEICS) in other countries such as the USA that considered as a standard for evaluating hospitals by the Joint Commission on Accreditation of Health Care Organizations (JCAHO).

## 6. Conclusion

Proper management of hospitals as organizations with an increased demand for, in the time of crisis, requires much more of both theoretical and practical abilities and skills. Hence, it is essential for any hospital, based on their resources, opportunities, risks and specific characteristics, to have a specific crisis management plan in general and a particular design for hospital preparedness against emergencies such as earthquake, to guarantee the hospital’s adequate performance during a crisis.

Our results show that, the studied hospital preparedness against earthquake is relatively high. However, about half of the investigated hospital buildings were over 30 years old and along with the wanted or unwanted changes that could arise in different areas of them, prevention of diminution of preparedness but also improvement of it should be a top priority of managers. Furthermore, continuous observance and periodic control of hospital managers’ performances regarding executing instructions of “Planning Guide for Hospitals to deal with quake”, and removal of possible defects should always be priorities of the top managers of the medical sciences university. This study has potential implications to hospital policies/practices. Healthcare managers in local and national levels can provide appropriate infrastructure for enhancing preparedness of hospitals against earthquake. This can be conducted through emphasizing on more important structural and nonstructural dimensions of safety. It recommended that future studies noticed other dimensions of hospital preparedness against earthquake. In order to designing comprehensive and more deep study, use of other methodologies, including qualitative researches and considering of other disaster in the health care environment would be helpful.
